# Self-Harm and Suicide Rates Before and After an Early Intervention Program for Patients With First-Episode Schizophrenia

**DOI:** 10.1001/jamanetworkopen.2024.26795

**Published:** 2024-08-08

**Authors:** Yi Chai, Jennifer Yee-Man Tang, Dennis Chak Fai Ma, Hao Luo, Sherry Kit Wa Chan

**Affiliations:** 1 Department of Pharmacology and Pharmacy, LKS Faculty of Medicine, The University of Hong Kong, Hong Kong SAR, China; 2 The Hong Kong Jockey Club Centre for Suicide Research and Prevention, The University of Hong Kong, Hong Kong SAR, China; 3 Department of Educational Psychology, The Chinese University of Hong Kong, Hong Kong SAR, China; 4 Sau Po Centre on Ageing, The University of Hong Kong, Hong Kong SAR, China; 5 Department of Nursing, The Hong Kong Polytechnic University, Hong Kong SAR, China; 6 Department of Psychiatry, School of Clinical Medicine, LKS Faculty of Medicine, The University of Hong Kong, Hong Kong SAR, China; 7 Department of Social Work and Social Administration, The University of Hong Kong, Hong Kong SAR, China; 8 State Key Laboratory of Brain and Cognitive Sciences, The University of Hong Kong, Hong Kong SAR, China

## Abstract

**Importance:**

Evidence on the association of early intervention services (EISs) with self-harm and suicide among patients with first-episode schizophrenia (FES) at older than 25 years is lacking.

**Objective:**

To examine changes in self-harm and suicide rates among patients with FES before and after the implementation of an EIS program.

**Design, Setting, and Participants:**

This population-based cohort study conducted among 37 040 patients aged 15 to 64 years with FES between January 1, 2001, and March 31, 2020, used electronic medical records from the Hong Kong Clinical Data Analysis and Reporting System. All patients were followed up from the first diagnosis of schizophrenia (the index date) until the date of their death or the end of the study period (March 31, 2021), whichever came first. Statistical analysis was performed from July to November 2023.

**Exposure:**

The EIS extended the Early Assessment Service for Young People With Early Psychosis (EASY) program from patients aged 15 to 25 years to those aged 15 to 64 years (EASY Plus). The exposure was the implementation of the EASY Plus program in April 2011. The exposure period was defined as between April 2012 and March 2021 for the 1-year-time-lag analysis.

**Main Outcomes and Measures:**

The outcomes were monthly rates of self-harm and suicide among patients with FES before and after the implementation of the EASY Plus program. Interrupted time series analysis was used for the main analysis.

**Results:**

This study included 37 040 patients with FES (mean [SD] age at onset, 39 [12] years; 82.6% older than 25 years; 53.0% female patients). The 1-year-time-lag analysis found an immediate decrease in self-harm rates among patients aged 26 to 44 years (rate ratio [RR], 0.77 [95% CI, 0.59-1.00]) and 45 to 64 years (RR, 0.70 [95% CI, 0.49-1.00]) and among male patients (RR, 0.71 [95% CI, 0.56-0.91]). A significant long-term decrease in self-harm rates was found for all patients with FES (patients aged 15-25 years: RR, 0.98 [95% CI, 0.97-1.00]; patients aged 26-44 years: RR, 0.98 [95% CI, 0.97-0.99]; patients aged 45-64 years: RR, 0.97 [95% CI, 0.96-0.98]). Suicide rates decreased immediately after the implementation of the EASY Plus program among patients aged 15 to 25 years (RR, 0.33 [95% CI, 0.14-0.77]) and 26 to 44 years (RR, 0.38 [95% CI, 0.20-0.73]). Compared with the counterfactual scenario, the EASY Plus program might have led to 6302 fewer self-harm episodes among patients aged 26 to 44 years.

**Conclusions and Relevance:**

This cohort study of the EASY Plus program suggests that the extended EIS was associated with reduced self-harm and suicide rates among all patients with FES, including those older than 25 years. These findings emphasize the importance of developing tailored interventions for patients across all age ranges to maximize the benefits of EISs.

## Key Points

**Question:**

Are early intervention services (EISs) associated with decreases in self-harm and suicide risk among patients older than 25 years with first-episode schizophrenia (FES)?

**Findings:**

In this population-based cohort study of 37 040 patients with FES, immediate and long-term decreases in self-harm were observed among patients older than 25 years after the implementation of the EIS. Suicide rates decreased immediately among patients aged 15 to 44 years.

**Meaning:**

This study suggests that the EIS was associated with decreases in self-harm and suicide among patients with FES, underscoring the importance of designing and implementing EISs for patients across all age ranges.

## Teaser

This 20-year cohort study examines changes in self-harm and suicide rates among patients older than 25 years with first-episode schizophrenia before and after the implementation of an early intervention services program.

## Introduction

Patients with schizophrenia are at increased risk of self-harm, suicide, and all-cause mortality,^1,2,3,4^ as well as a reduction of life expectancy of up to 20 years compared with the general population.^5^ The lifetime prevalence of self-harm among patients with schizophrenia is around 26.8%, and approximately 5.6% of individuals with schizophrenia die by suicide.^6,7^ A meta-analysis indicates that the median standardized mortality ratio for suicide among patients with schizophrenia is 12.86 (range, 0.66-174.25).^3^ The risk of self-harm and suicide during the early phase of schizophrenia is twice that during the later course of the illness, highlighting the importance of early intervention for first-episode schizophrenia (FES).^7,8^

In the past 2 decades, early intervention services (EISs) for patients with first-episode psychosis have been implemented in different locations,^9,10,11^ with demonstrable effectiveness in optimizing clinical and functional outcomes, including reductions in self-harm and suicide.^9,10,12,13,14^ However, most EISs focus on younger patients, with a maximum age limit ranging from 25 years in Australia to 45 years in Denmark and the US.^15,16,17^ Consequently, between 21% and 71% of patients with first-episode psychosis do not meet the age-entry criteria for most EISs, despite having similar or even higher care needs than younger patients.^12,16,18^ In response, the National Institute for Health and Care Excellence guidelines were updated to recommend expansion of the EIS to patients aged up to 60 years in the UK in 2014.^19,20^ In Hong Kong, the EIS was extended to patients aged up to 64 years in 2011.^21^ To our knowledge, these 2 programs are the only territory-wide EISs that cover adults older than 45 years.

Patients with later-onset psychosis present with varying levels of psychosocial needs and clinical complexities, potentially resulting in unique prognosis patterns.^12,22^ For example, patients with later-onset psychosis had more systematic persecutory delusions, lower scores on affective flattening and social withdrawal,^23^ and better cognitive functions compared with those with early-onset psychosis.^24^ This finding suggests that the effectiveness of EISs in reducing self-harm and suicide may vary depending on the patient’s age at onset of illness.

However, studies examining EISs among the adult population, specifically when stratified by age groups, are limited,^10,25^ and to our knowledge, no study has examined the effectiveness of territory-wide EISs at the population level. Furthermore, most evidence on the effectiveness of EISs generally focuses on short-term observations,^10^ with few studies addressing long-term outcomes.^26^

Most current evidence on the effectiveness of EISs has been generated from randomized clinical trials (RCTs), providing good evidence on the effectiveness of the intervention for specific populations.^9^ However, these RCTs have limitations in generalizability and external validity and typically have shorter follow-up durations.^27,28,29^ Furthermore, the sample size of standard RCTs may be inadequate to evaluate associations of the intervention with rare outcomes such as self-harm and suicide.^30^ Our study, using clinical data, can therefore supplement existing RCT evidence and generate potential hypotheses regarding the effectiveness of EISs in reducing self-harm and suicide risks among patients with FES for future RCT validation.

The risk of self-harm and suicide may vary among patients with schizophrenia across groups with different ages at onset, although current evidence remains inconsistent. A systematic review suggested that older age at the onset of schizophrenia is associated with a higher risk of suicide,^31^ while other studies found that patients with an earlier onset are more likely to engage in self-harm and suicide behaviors.^6,32^ Beyond clinical interventions, rates of self-harm and suicide fluctuate over time and are closely related to societal changes, such as economic fluctuations and major events including disasters and infectious pandemics.^33,34^ Therefore, understanding the association of EISs that cover a wider age group with self-harm and suicide rates using population-based electronic medical records, adjusting for several covariates over a longer period, would provide important naturalistic and clinical evidence on the effect of EISs on patients older than 25 years. This investigation is crucial for future service and policy development.

In Hong Kong, the territory-wide EIS, the Early Assessment Service for Young People With Psychosis (EASY), was established in 2001 to provide a 2-year, phase-specific intervention for patients aged 15 to 25 years with first-episode psychosis.^14,35^ With the support of developing the EIS model for adults aged 26 to 55 years with first-episode psychosis in Hong Kong, based on a 4-year RCT involving 1100 participants,^36^ the EASY service was expanded in 2011 to a 3-year program for patients aged 15 to 64 years with first-episode psychosis (EASY Plus).^21^ Details of the EASY and EASY Plus programs are published elsewhere^35,37^ and provided in eAppendix 1 in Supplement 1. The present study uses population-based data to examine changes in monthly self-harm and suicide rates among patients with FES before and after the implementation of the expanded EIS, EASY Plus. Only patients with a schizophrenia diagnosis were examined because most participants in the EASY Plus program have a diagnosis of schizophrenia.^38^

## Methods

### Data Sources

This population-based cohort study used territory-wide electronic medical records from the Clinical Data Analysis and Reporting System (CDARS) in Hong Kong, which is managed by the Hong Kong Hospital Authority, a statutory body responsible for providing public hospital services, including the EASY Plus service, in Hong Kong.^39^ The Clinical Data Analysis and Reporting System has collected health care information, including demographic characteristics, clinical diagnoses, admission and discharge records, and prescriptions, across inpatient, outpatient, and accident and emergency departments since 1993.^40^ The diagnostic information in CDARS was stored in *International Classification of Diseases, Ninth Revision, Clinical Modification* (*ICD-9-CM*) codes. The Hospital Authority is the sole provider of inpatient psychiatric services in Hong Kong.^40^ The quality and reliability of CDARS data have been extensively validated by various epidemiologic studies, including those focusing on psychiatric outcomes.^14,41,42,43,44^ This study was approved by the institutional review board of the University of Hong Kong. Because all records were deidentified and no patients were contacted, the institutional review board waived informed consent. This study adhered to the Strengthening the Reporting of Observational Studies in Epidemiology (STROBE) reporting guideline.^45^

### Study Design and Sample Identification

Data on all patients who received their first diagnosis of schizophrenia (*ICD-9-CM* code 295) between January 1, 2001, and March 31, 2020, were retrieved from CDARS. These data were ascertained by examining previous diagnoses in CDARS records between January 1, 1993 (the earliest available CDARS data), and December 31, 2000. The date of the first diagnosis of schizophrenia during the study period was designated as the index date. All patients were followed up from the first diagnosis of schizophrenia (the index date) until the date of their death or the end of the study period (March 31, 2021), whichever came first.

Patients were excluded if they had missing values for sex, date of birth, or date of death; were younger than 15 years or older than 64 years; had incorrect records; had less than 1 year of follow-up; or had no records after the index date. eFigure 1 in Supplement 1 shows the flowchart of the study sample selection.

### Exposure and Outcomes

The exposure was the EASY Plus program, implemented in April 2011. Given that the EASY Plus program may take time to take effect, a 1-year time lag was introduced in the main analysis. Specifically, the 1-year period (April 2011 to March 2012) after the introduction of the EASY Plus program was considered a transitional period and excluded from the analysis. The study period was divided into 2 phases: the pre–EASY Plus period (January 2001-March 2011) and the EASY Plus period (April 2012-March 2021). Specifically, the pre–EASY Plus period refers to the period when a 2-year EASY service was available only for patients aged 15 to 25 years. Patients older than 25 years received standard psychiatric service as usual, and no EIS was available to them during this period. Further analysis was conducted without taking the time lag into account. For this analysis, the pre–EASY Plus period was designated as January 2001 to March 2011 and the EASY Plus period was designated as April 2011 to March 2021.

The outcomes in this study were monthly self-harm and suicide rates among patients with FES before and after the implementation of the EASY Plus program. Diagnoses of self-harm (such as self-poisoning, hanging, drowning, and cutting) were identified using the *ICD-9-CM* codes E950-959 and E980-989, which included both attempted suicide and deliberate self-harm without suicidal intent. Suicide (*International Statistical Classification of Diseases and Related Health Problems, Tenth Revision, Clinical Modification* [*ICD-10-CM*] codes X60-84 and Y10-34) was identified using cause of death information. All self-harm records, including both the first incidence of self-harm and repeated self-harms, after the first diagnosis of schizophrenia were identified.

### Covariates

Covariates included territory-wide standardized psychiatric inpatient beds per 100 000 people,^46^ the implementation of general community mental health services, social unrest events, and major pandemics throughout the study period. The detailed description of these covariates is shown in eAppendix 2 in Supplement 1.

### Statistical Analysis

Statistical analysis was performed from July to November 2023. Interrupted time series analysis using quasi-Poisson regression was used to examine the association of the EASY Plus program with self-harm and suicide rates among patients with FES. All parameters were expressed as rate ratios (RRs) with 95% CIs. A 2-sided *P* ≤ .05 was considered indicative of statistical significance. The number of all self-harm episodes or suicide cases in each month was included as the outcome variable, and the cumulative number of patients with a first diagnosis of schizophrenia from January 2001 up to that specific month was included as an offset variable to quantify the monthly rates of self-harm and suicide. Data from the first 2 calendar years (2001 and 2002) were excluded from the interrupted time series analysis because, at the beginning of the study period, the cumulative number of patients with FES (denominator) was relatively small, leading to potentially biased estimation of monthly rates of self-harm and suicide. Time in months since January 2003 was included as a continuous variable to measure the time effect. The intervention variable, which was set as 0 for the pre–EASY Plus period and as 1 for the EASY Plus period, was included to represent the level change (immediate change). An interaction term between the intervention variable and time since the beginning of the EASY Plus period (April 2011 or April 2012) was included to represent the slope change (long-term change). Calendar time was included as a categorical variable divided into 4 seasons: spring (March-May), summer (June-August), autumn (September-November), and winter (December-February), as defined by the Hong Kong Observatory, to control for seasonality.^47^ The autocorrelation was accounted for by including first-order lagged residuals.^48^

A counterfactual estimation was generated, assuming that no intervention had been implemented, to quantify the absolute change in the number of self-harm episodes and suicide cases after the implementation of EASY Plus. In this scenario, the intervention term was set to 0 throughout the entire period. The absolute differences in terms of all self-harm episodes and suicide cases were calculated by comparing the interrupted time series model–fitted values and counterfactual values.

All analyses were stratified by gender and age groups (15-25, 26-44, and 45-64 years). Three additional sets of sensitivity analyses were conducted, including a 2-year-time-lag analysis, in which the EASY Plus period was defined as April 2013 to March 2021; a calculation of Newey-West SEs to correct for possible autocorrelation^49^; and an analysis based on stricter definition of self-harm (*ICD-9-CM* codes E950-959) and suicide (*ICD-10-CM* codes X60-84), to test the robustness of the results. All analyses were performed using R software, version 4.1.2 (R Project for Statistical Computing).^50^

## Results

The overall study sample comprised 37 040 patients with FES between January 2001 and March 2020 (mean [SD] age at onset, 39 [12] years; 82.6% older than 25 years; 53.0% female patients). The mean (SD) annual number of patients aged 15 to 64 years with an incident diagnosis of schizophrenia within CDARS was 1545 (266.9) since the implementation of EASY Plus (2011). The overall self-harm rate was 15.9% (95% CI, 15.5%-16.3%) and the overall suicide rate was 1.7% (95% CI, 1.6%-1.8%) (Table 1) between January 2003 and March 2021. The highest self-harm and suicide rates during the study period were observed among patients aged 15 to 25 years (self-harm, 23.0% [95% CI, 21.8%-24.2%]; suicide, 2.5% [2.2%-3.0%]). Patients aged 45 to 64 years had the lowest self-harm and suicide rates (self-harm, 10.0% [95% CI, 9.5%-10.5%]; suicide, 1.0% [95% CI, 0.9%-1.2%]) during the study period.

**Table 1.** Self-Harm and Suicide Rates Among Patients With FES Between January 2003 and March 2021

Characteristic	No. of patients with FES (%)	Self-harm		Suicide	
		No. of events	Rate, % (95% CI)	No. of events	Rate, % (95% CI)
All patients	37 040 (100)	5900	15.9 (15.5-16.3)	632	1.7 (1.6-1.8)
Age, y					
15-25	6452 (17.4)	1482	23.0 (21.8-24.2)	164	2.5 (2.2-3.0)
26-44	17 065 (46.1)	3068	18.0 (17.4-18.6)	331	1.9 (1.7-2.2)
45-64	13 523 (36.5)	1350	10.0 (9.5-10.5)	137	1.0 (0.9-1.2)
Sex					
Male	17 415 (47.0)	3486	20.0 (19.4-20.7)	370	2.1 (1.9-2.4)
Female	19 625 (53.0)	2414	12.3 (11.8-12.8)	262	1.3 (1.2-1.5)

Abbreviation: FES, first-episode schizophrenia.

**Figure 1.**
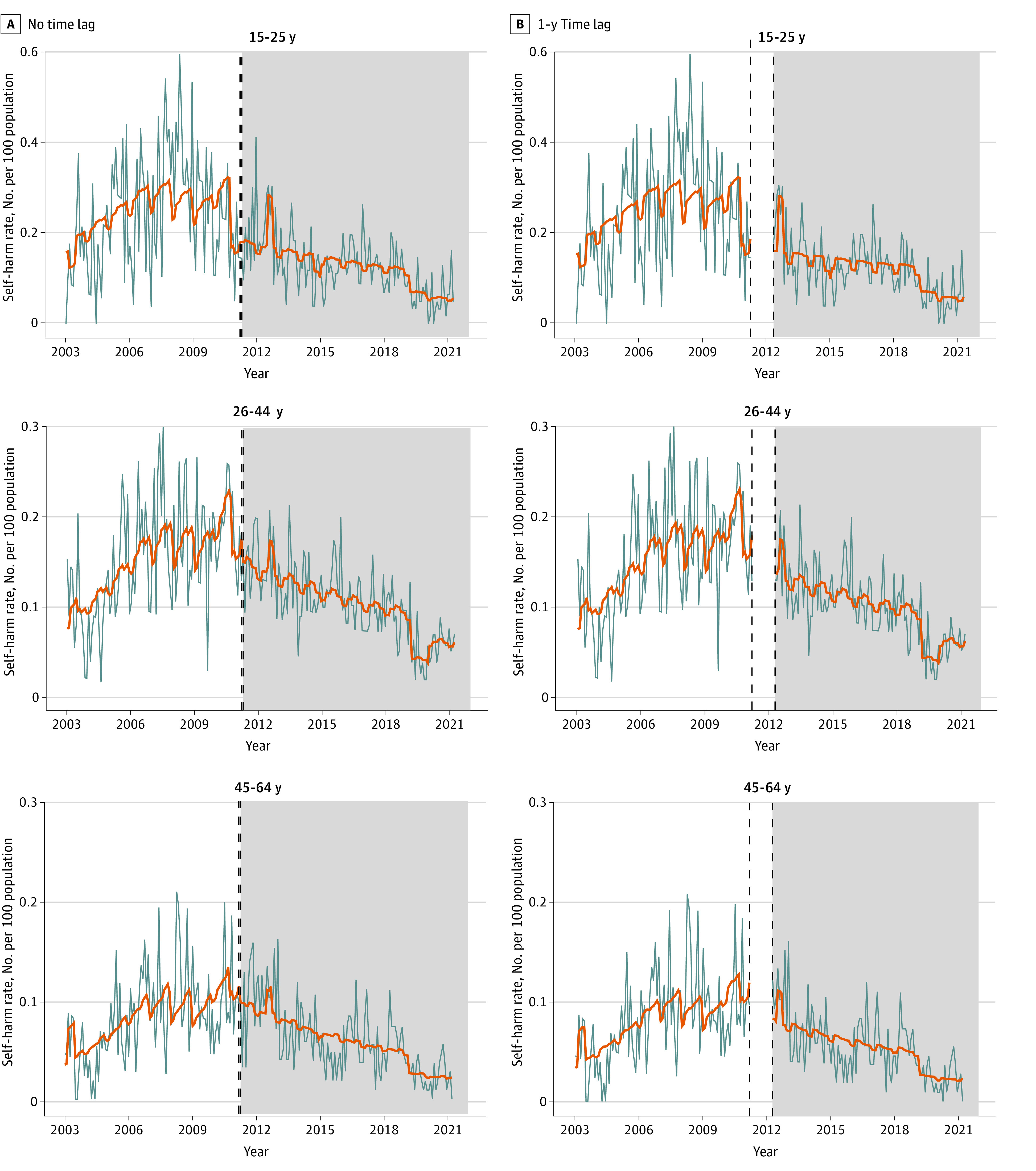
Interrupted Time Series Analysis of Changes in Self-Harm Rates by Age Group Before and After the Implementation of the Early Assessment Service for Young People With Early Psychosis for Patients Aged 15 to 64 Years (EASY Plus) Program Gray lines indicate the observed self-harm rates. Orange lines indicate the fitted self-harm rates based on the interrupted time series model. Gray areas indicate the EASY Plus intervention time. Vertical dashed lines in the no time lag analysis indicate March and April 2011. Vertical dashed lines in the 1-year-time-lag analysis indicate March 2011 and March 2012.

**Figure 2.**
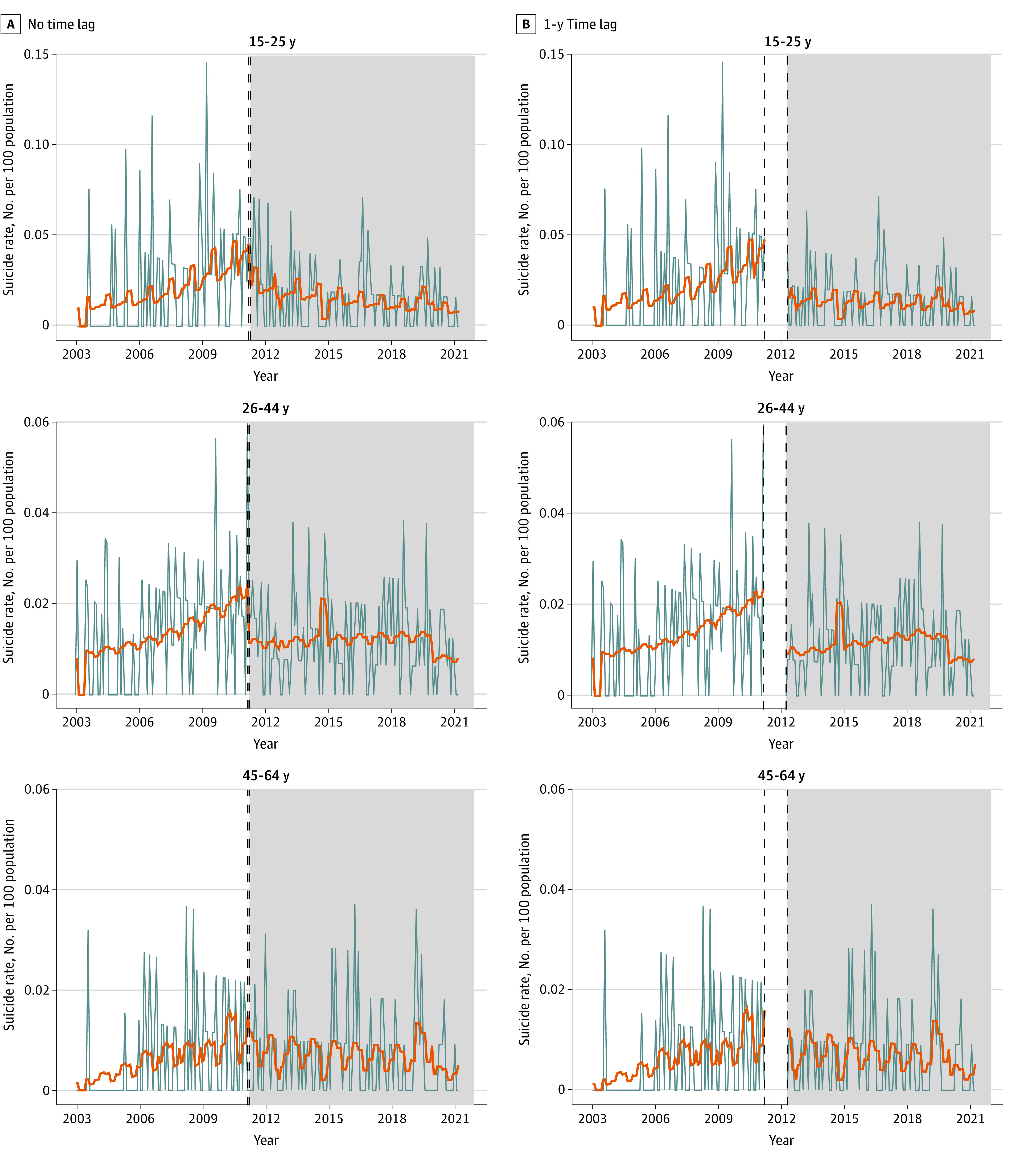
Interrupted Time Series Analysis of Changes in Suicide Rates by Age Group Before and After the Implementation of the Early Assessment Service for Young People With Early Psychosis for Patients Aged 15 to 64 Years (EASY Plus) Program Gray lines indicate the observed suicide rates. Orange lines indicate the fitted suicide rates based on the interrupted time series model. Gray areas indicate the EASY Plus intervention time. Vertical dashed lines in the no time lag analysis indicate March and April 2011. Vertical dashed lines in the 1-year-time-lag analysis indicate March 2011 and March 2012.

Figures 1 and 2 and eFigures 2 and 3 in Supplement 1 show the observed monthly rates of self-harm and suicide and the fitted values obtained from interrupted time series analyses of patients with FES before and during the EASY Plus program intervention. Table 2 shows the parameter estimates of level (immediate) and slope (long-term) changes in self-harm and suicide rates after the EASY Plus intervention.

**Table 2.** Estimates of the Level and Slope Changes in Self-Harm and Suicide Rates Among Patients With First-Episode Schizophrenia After the Implementation of the EASY Plus Program

Characteristic	Rate ratio (95% CI)			
	No time lag		One-year time lag	
	Level change	Slope change	Level change	Slope change
**Self-harm**				
Age, y				
15-25	0.99 (0.65-1.52)	0.98 (0.97-1.00)^a^	0.86 (0.56-1.33)	0.98 (0.97-1.00)^a^
26-44	0.84 (0.65-1.08)	0.98 (0.97-0.98)	0.77 (0.59-1.00)^a^	0.98 (0.97-0.99)
45-64	0.84 (0.59-1.18)	0.97 (0.95-0.98)	0.70 (0.49-1.00)^a^	0.97 (0.96-0.98)
Sex				
Male	0.83 (0.65-1.05)	0.98 (0.97-0.98)	0.71 (0.56-0.91)	0.98 (0.97-0.98)
Female	0.92 (0.69-1.24)	0.98 (0.98-0.99)	0.87 (0.64-1.18)	0.98 (0.97-0.99)
**Suicide**				
Age, y				
15-25	0.51 (0.23-1.10)	0.98 (0.95-1.02)	0.33 (0.14-0.77)	0.99 (0.95-1.03)
26-44	0.47 (0.26-0.85)	1.00 (0.97-1.02)	0.38 (0.20-0.73)	1.00 (0.97-1.03)
45-64	0.75 (0.26-2.12)	0.96 (0.92-1.01)	0.78 (0.27-2.23)	0.96 (0.71-1.00)
Sex				
Male	0.47 (0.26-0.85)	0.98 (0.96-1.01)	0.43 (0.23-0.80)	0.99 (0.96-1.01)
Female	0.61 (0.32-1.17)	0.98 (0.96-1.01)	0.45 (0.23-0.88)	0.99 (0.96-1.02)

Abbreviation: EASY Plus, Early Assessment Service for Young People With Early Psychosis for Patients Aged 15 to 64 Years.

^a^ The *P* value of the rate ratio is less than .05.

In the 1-year-time-lag analysis, a significant immediate decrease in self-harm rates was observed among patients aged 26 to 44 years (RR, 0.77 [95% CI, 0.59-1.00]) and 45 to 64 years (RR, 0.70 [95% CI, 0.49-1.00]) and among male patients (RR, 0.71 [95% CI, 0.56-0.91]) (Table 2). A significant long-term decrease in self-harm rates was found for all patients with FES (patients aged 15-25 years: RR, 0.98 [95% CI, 0.97-1.00]; patients aged 26-44 years: RR, 0.98 [95% CI, 0.97-0.99]; patients aged 45-64 years: RR, 0.97 [95% CI, 0.96-0.98]). One year after the implementation of EASY Plus, suicide rates decreased immediately among patients aged 15 to 25 years (RR, 0.33 [95% CI, 0.14-0.77]) and 26 to 44 years (RR, 0.38 [95% CI, 0.20-0.73]) and both male (RR, 0.43 [95% CI, 0.23-0.80]) and female patients (RR, 0.45 [95% CI, 0.23-0.88]).

The results from the no time-lag analysis were generally consistent with the 1-year-time-lag analysis, although the immediate changes were mostly not significant. eTables 1 to 4 in Supplement 1 show the full results from the interrupted time series analyses of self-harm and suicide rates.

Table 3 shows the cumulative number of differences in self-harm episodes between fitted and counterfactual values since the implementation of EASY Plus. The potential reductions in self-harm episodes or suicide cases during the intervention period were only estimated for groups with significant slope changes (long-term changes), as per Table 2. According to the 1-year-time-lag analysis, implementing the EASY Plus program was associated with a reduction of 1314 self-harm episodes among patients aged 15 to 25 years (reductions per 100 patients, 20 [95% CI, 19-21]), 6302 self-harm episodes among patients aged 26 to 44 years (reductions per 100 patients, 37 [95% CI, 36-38]), and 4711 self-harm episodes among patients aged 45 to 64 years (reductions per 100 patients, 35 [95% CI, 34-36]) (Table 3). Sensitivity analyses, which included a 2-year-time-lag analysis, the Newey-West SEs correction, and an analysis based on stricter definitions of self-harm and suicide, did not alter the main findings (eTables 5 and 6 in Supplement 1).

**Table 3.** Estimated Reduction in the Number of Self-Harm Episodes Since the Implementation of the EASY Plus Program

Time lag	No. of patients	Fitted No. of self-harm episodes (95% CI)^a^	Counterfactual No. of self-harm episodes (95% CI)^b^	Cumulative No. of differences in self-harm episodes since April 1, 2011, or April 1, 2012	Difference in fitted and counterfactual No. of self-harm episodes per 100 population (95% CI)
**No time lag**					
Age, y					
15-25	6452	1485 (1205-1846)	3211 (1486-8582)	1726	27 (26-28)
26-44	17 065	3067 (2656-3555)	11 465 (5849-24 923)	8398	49 (48-50)
45-64	13 523	1353 (1099-1681)	7920 (2870-26 611)	6567	49 (47-50)
Sex					
Male	17 415	3487 (3045-4006)	13 139 (7046-26 821)	9652	55 (54-57)
Female	19 625	2418 (2064-2846)	7781 (3860-17 725)	5363	27 (27-28)
**1-y Time lag**					
Age, y					
15-25	6452	1387 (1119-1732)	2701 (1390-6196)	1314	20 (19-21)
26-44	17 065	2852 (2452-3330)	9154 (5073-18 058)	6302	37 (36-38)
45-64	13 523	1238 (995-1552)	5949 (2477-16 938)	4711	35 (34-36)
Sex					
Male	17 415	3225 (2808-3716)	10312 (6094-18 769)	7087	41 (40-42)
Female	19 625	2251 (1906-2670)	6268 (3386-12 887)	4017	20 (20-21)

Abbreviation: EASY Plus, Early Assessment Service for Young People With Early Psychosis for Patients Aged 15 to 64 Years.

^a^ With the EASY Plus program.

^b^ Without the EASY Plus program.

## Discussion

To our knowledge, this is the first study to examine the association of EISs with self-harm and suicide among patients older than 25 years with FES using population-based data. We found significant immediate decreases in self-harm rates among patients older than 25 years with a 1-year time lag. A long-term reduction in self-harm rates was observed for all patients. In addition, in the main analysis, the immediate decreases in suicide rates were significant for patients aged 15 to 44 years but not for those older than 45 years, and the monthly suicide death rates remained stable without further significant reduction. These findings provided empirical evidence highlighting the importance of tailoring EISs to patients in different age groups with FES.

As the territory-wide EIS, the continuous support and psychosocial interventions provided by key workers as part of the EASY Plus program may improve coping strategies and problem-solving skills and facilitate timely risk identification and intervention, thus reducing self-harm and suicide risks. However, these findings are derived from population-level observations using aggregated monthly data, without accounting for individual-level risk factors of self-harm and suicide. Therefore, these reductions should be interpreted at the macro level. Furthermore, the most significant results observed in the 1-year-time-lag analyses suggested that there may be a necessary period for effectively implementing EISs, including the establishment of adequate manpower and training.

Our study found an overall prevalence of self-harm at 15.9% and a prevalence of suicide at 1.7% between January 2003 and March 2021. These rates were similar to previous estimates from the Chinese population^51^ but different from those reported in other countries.^6,7,52,53,54,55,56^ Several factors may be associated with these discrepancies. First, the information on self-harm and suicide was obtained from electronic medical records, which can be subject to underrepresentation due to documentation accuracy. Second, in many Asian societies, including Hong Kong, there is a strong stigma associated with mental illness, which may result in both individuals’ reluctance to report suicidal behaviors and underreporting in medical records.^57^ In addition, different population characteristics, diagnostic criteria, and follow-up periods can also influence the reported results. Therefore, caution is required when comparing our self-harm and suicide rates with findings from other studies.

Most EISs internationally focus on the younger population, with few targeting the older population. Previous studies have highlighted the different treatment outcomes of EISs, including disengagement patterns, among patients of different ages with FES.^21^ To our knowledge, no previous study has evaluated the association of EISs with self-harm and suicide among a population older than 25 years. Our results showed a significant immediate reduction in self-harm rates among patients older than 25 years with FES after the introduction of the EASY Plus program with a 1-year time lag. A long-term reduction in self-harm rates was observed for all patients. A possible explanation is that the 2-year EASY program has been already implemented for the 15- to 25-year-old age group since 2001. The only difference between EASY Plus and the EASY service was the service duration, which may explain the lack of significant immediate changes in self-harm rates. However, the significant continued reductions among the 15- to 25-year-old age group suggests sustained benefits associated with the extended EASY program among youths.

A significant immediate reduction in suicide rates was observed for patients younger than 45 years after the implementation of the EASY Plus program. One possibility is that the suicide rate in the 45- to 64-year-old age group was low (1.0%), which made the model not sufficiently powered to detect significant changes. In addition, according to the consensus definition, patients with schizophrenia with an onset age of 45 to 64 years are categorized as having late-onset schizophrenia (>40 years) or very-late-onset schizophrenia (>60 years).^58^ Patients with late-onset and very-late-onset schizophrenia may exhibit different clinical presentations and causes compared with those with earlier-onset schizophrenia, thereby representing distinct service needs.^23,24^ For example, due to the higher prevalence of comorbidity and age-specific issues, patients with late-onset and very-late-onset schizophrenia may require comprehensive physical assessment, better medication management, and customized psychosocial intervention.^12^ The differential associations of the EIS with self-harm and suicide for different age groups highlight the importance of tailoring services to meet the diverse needs of patients with different demographic characteristics.

### Limitations

Although our findings are based on a population-based dataset, the study has several limitations. First, although the EASY Plus program is a territory-wide intervention, it currently can be provided to only 1300 new clients per year due to workload constraints. However, our preliminary findings showed that the mean annual number of patients aged 15 to 64 years within CDARS with incident schizophrenia since 2011 was 1545, indicating that most patients with FES should have received the EASY Plus service. Consequently, the association of EASY Plus with self-harm and suicide, if any, should be evident at the population level. Second, the accuracy of electronic medical records depends on the input of clinicians, which might affect the timing of the diagnosis input. Moreover, some patients may receive their first diagnosis of schizophrenia outside Hong Kong. Therefore, patients with FES included in this study may not truly be new cases for the specific year. In addition, some self-harm episodes might not be reported to clinicians or be documented, potentially limiting the reliability of the results. Third, the EASY Plus program may not be the sole factor associated with the observed decreased risks of self-harm and suicide. Although we have controlled for the initiation of major community mental health services, we were unable to include other services and relevant social policies. Furthermore, apart from the social determinants of self-harm and suicide considered in this study, other factors such as economic changes reflected by unemployment rates, gross domestic product, inflation rate, changes in population characteristics due to immigration, and the Werther effect (increased suicide rates after well-publicized reports of deaths by suicide of celebrities or other well-known figures in the media) and Papageno effect (decreased suicide rates after mass media promotes alternatives to suicide) from media reporting were not included. These factors might lead to a biased estimation of the reduction in self-harm and suicide associated with the EASY Plus program. Fourth, given the anonymity of the data, it was not possible to validate the causes of death with external sources, including the Coroner’s Court Report, thus introducing possible bias to the results. Fifth, this study examined the association of EASY Plus with self-harm and suicide rates at a population level. Limited by the study design, we were not able to account for individual-level factors associated with self-harm and suicide, such as functional characteristics, socioeconomic status, psychiatric comorbidities, interventions received, and compliance.

## Conclusions

This population-based cohort study suggests that the expanded EIS (EASY Plus) was associated with lower long-term self-harm and suicide rates among all patients with FES, including those older than 25 years. Our results highlight the importance of tailoring the EIS to meet the unique needs of diverse patient subgroups when developing and implementing EISs for a broader adult population.

## Notes

Supplement 1. **eAppendix 1.** Detailed Description of EASY and EASY Plus Programs
eReferences 1.
**eAppendix 2.** Detailed Description of Covariates
eReferences 2.
**eFigure 1.** Flowchart of the Study Sample Selection **eFigure 2.** Interrupted Time Series Analysis of Changes in Self-Harm Rates by Sex Group Before and After the Implementation of the Early Assessment Service for Young People With Early Psychosis for Patients Aged 15 to 64 Years (EASY Plus) Program **eFigure 3.** Interrupted Time Series Analysis of Changes in Suicide Rates by Sex Group Before and After the Implementation of the Early Assessment Service for Young People With Early Psychosis for Patients Aged 15 to 64 Years (EASY Plus) Program **eTable 1.** Full Results From Interrupted Time Series Analyses of Self-Harm Rates for the No-Time-Lag Analysis **eTable 2.** Full Results From Interrupted Time Series Analyses of Self-Harm Rates for the One-Year-Time-Lag Analysis **eTable 3.** Full Results From the Interrupted Time Series Analysis of Suicide Rates for the No-Time-Lag Analysis **eTable 4.** Full Results From the Interrupted Time Series Analysis of Suicide Rates for the One-Year-Time-Lag Analysis **eTable 5.** Estimates of the Level and Slope Changes in Self-Harm and Suicide Rates From the Two-Year-Time-Lag Analysis and Newey-West Standard Errors Corrected Analysis **eTable 6.** Estimates of the Level and Slope Changes in Self-Harm and Suicide Rates From the Stricter Definition of Self-Harm and Suicide Analysis

Supplement 2.
Data Sharing Statement
