# Supplementary material for: Self-Harm and Suicide Rates Before and After an Early Intervention Program for Patients With First-Episode Schizophrenia
Source: JAMA Netw Open. 2024 Aug 8;7(8):e2426795. doi: 10.1001/jamanetworkopen.2024.26795 (PMC11310822; doi:10.1001/jamanetworkopen.2024.26795)
Supplement: Supplement 1. — eAppendix 1. Detailed Description of EASY and EASY Plus Programs eReferences 1. eAppendix 2. Detailed Description of Covariates eReferences 2. eFigure 1. Flowchart of the Study Sample Selection eFigure 2. Interrupted Time Series Analysis of Changes in Self-Harm Rates by Sex Group Before and After the Implementation of the Early Assessment Service for Young People With Early Psychosis for Patients Aged 15 to 64 Years (EASY Plus) Program eFigure 3. Interrupted Time Series Analysis of Changes in Suicide Rates by Sex Group Before and After the Implementation of the Early Assessment Service for Young People With Early Psychosis for Patients Aged 15 to 64 Years (EASY Plus) Program eTable 1. Full Results From Interrupted Time Series Analyses of Self-Harm Rates for the No-Time-Lag Analysis eTable 2. Full Results From Interrupted Time Series Analyses of Self-Harm Rates for the One-Year-Time-Lag Analysis eTable 3. Full Results From the Interrupted Time Series Analysis of Suicide Rates for the No-Time-Lag Analysis eTable 4. Full Results From the Interrupted Time Series Analysis of Suicide Rates for the One-Year-Time-Lag Analysis eTable 5. Estimates of the Level and Slope Changes in Self-Harm and Suicide Rates From the Two-Year-Time-Lag Analysis and Newey-West Standard Errors Corrected Analysis eTable 6. Estimates of the Level and Slope Changes in Self-Harm and Suicide Rates From the Stricter Definition of Self-Harm and Suicide Analysis [file jamanetwopen-e2426795-s001.pdf]

Supplementary Online Content

Chai Y, Tang JYM, Ma DCF, Luo H, Chan SKW. Self-harm and suicide rates before and after an early intervention program for patients with first-episode schizophrenia. *JAMA Netw Open.* 2024;7(8):e2426795. doi:10.1001/jamanetworkopen.2024.26795

eAppendix 1. Detailed Description of EASY and EASY Plus Programs
eReferences 1.
eAppendix 2. Detailed Description of Covariates
eReferences 2.
eFigure 1. Flowchart of the Study Sample Selection
eFigure 2. Interrupted Time Series Analysis of Changes in Self-Harm Rates by Sex Group Before and After the Implementation of the Early Assessment Service for Young People With Early Psychosis for Patients Aged 15 to 64 Years (EASY Plus) Program
eFigure 3. Interrupted Time Series Analysis of Changes in Suicide Rates by Sex Group Before and After the Implementation of the Early Assessment Service for Young People With Early Psychosis for Patients Aged 15 to 64 Years (EASY Plus) Program
eTable 1. Full Results From Interrupted Time Series Analyses of Self-Harm Rates for the No-Time-Lag Analysis
eTable 2. Full Results From Interrupted Time Series Analyses of Self-Harm Rates for the One-Year-Time-Lag Analysis
eTable 3. Full Results From the Interrupted Time Series Analysis of Suicide Rates for the No-Time-Lag Analysis
eTable 4. Full Results From the Interrupted Time Series Analysis of Suicide Rates for the One-Year-Time-Lag Analysis
eTable 5. Estimates of the Level and Slope Changes in Self-Harm and Suicide Rates From the Two-Year-Time-Lag Analysis and Newey-West Standard Errors Corrected Analysis
eTable 6. Estimates of the Level and Slope Changes in Self-Harm and Suicide Rates From the Stricter Definition of Self-Harm and Suicide Analysis

This supplementary material has been provided by the authors to give readers additional information about their work.

© 2024 Chai Y et al. *JAMA Network Open.*

eAppendix 1. Detailed Description of EASY and EASY Plus Programs

The EASY service is a phase-specific multidisciplinary service provided by clinicians and key workers to patients with first-episode psychosis (FEP) based on a specifically developed protocol according to the international consensus of early intervention for psychosis (Bertolote & McGorry 2005). This protocol focuses on initial engagement, psychoeducation, psychological adjustment to the illness, and management of comorbidities, with pharmacological treatment tailored to the stages and needs of patients. Vocational training and psychosocial rehabilitation for patients were also coordinated in collaboration with other service providers (Chan et al., 2015; Tang et al., 2010). The EASY service for FEP patients aged 15-25, implemented in 2011, was 2 years in duration. In 2011, the service was extended (EASY plus) to serve patients with FEP aged 15-64 for 3 years, with the key service components remaining the same. Prior to EASY plus, FEP patients aged over 25 received standard adult psychiatric service, which largely included inpatient and outpatient psychiatric clinic services and community psychiatric nurse services for management of risk without specific coordination (Chan et al., 2015).

eReferences 1.

Bertolote, J., & McGorry, P. (2005, Aug). Early intervention and recovery for young people with early psychosis: consensus statement. *Br J Psychiatry Suppl, 48*, s116-119. https://doi.org/10.1192/bjp.187.48.s116

Chan, S. K., So, H. C., Hui, C. L., Chang, W. C., Lee, E. H., Chung, D. W., Tso, S., Hung, S. F., Yip, K. C., Dunn, E., & Chen, E. Y. (2015, Apr). 10-year outcome study of an early intervention program for psychosis compared with standard care service. *Psychol Med, 45*(6), 1181-1193. https://doi.org/10.1017/s0033291714002220

Tang, J. Y. M., Wong, G. H. Y., Hui, C. L. M., Lam, M. M. L., Chiu, C. P. Y., Chan, S. K. W., Chung, D. W. S., Tso, S., Chan, K. P. M., Yip, K. C., Hung, S. F., & Chen, E. Y. H. (2010). Early intervention for psychosis in Hong Kong – the EASY programme. *Early Intervention in Psychiatry, 4*(3), 214-219. https://doi.org/https://doi.org/10.1111/j.1751-7893.2010.00193.x

© 2024 Chai Y et al. *JAMA Network Open.*

eAppendix 2. Detailed Description of Covariates

The general community mental health service refers to the territory-wide Integrated Community Centre for Mental Wellness (ICCMW) program, operated by non-government organizations funded by the Social Welfare Department of Hong Kong to provide community mental health services for patients with mental health conditions and their family members since October 2010 (Ma et al., 2023) Three large social unrest events lasting for three months or more in Hong Kong were identified, the Moral and National Education Controversy in 2012 (July-September), the Umbrella Movement in 2014 (September-December), and the Anti-extradition Law Amendment Bill Movement in 2019 (March-December) (Ma et al., 2023). A major pandemic period was defined as the period when infectious diseases spread across a large geographical area within a short timeframe. In this study, two major pandemics were identified, the SARS (March-June 2003) and COVID-19 (January 2020-March 2021) outbreaks.

eReferences 2.

Ma, C. F., Luo, H., Leung, S. F., Wong, G. H. Y., Lam, R. P. K., Bastiampillai, T., Chen, E. Y. H., & Chan, S. K. W. (2023). Impact of community mental health services on the adult psychiatric admission through the emergency unit: a 20-year population-based study. The Lancet Regional Health – Western Pacific. https://doi.org/10.1016/j.lanwpc.2023.100814

© 2024 Chai Y et al. *JAMA Network Open.*

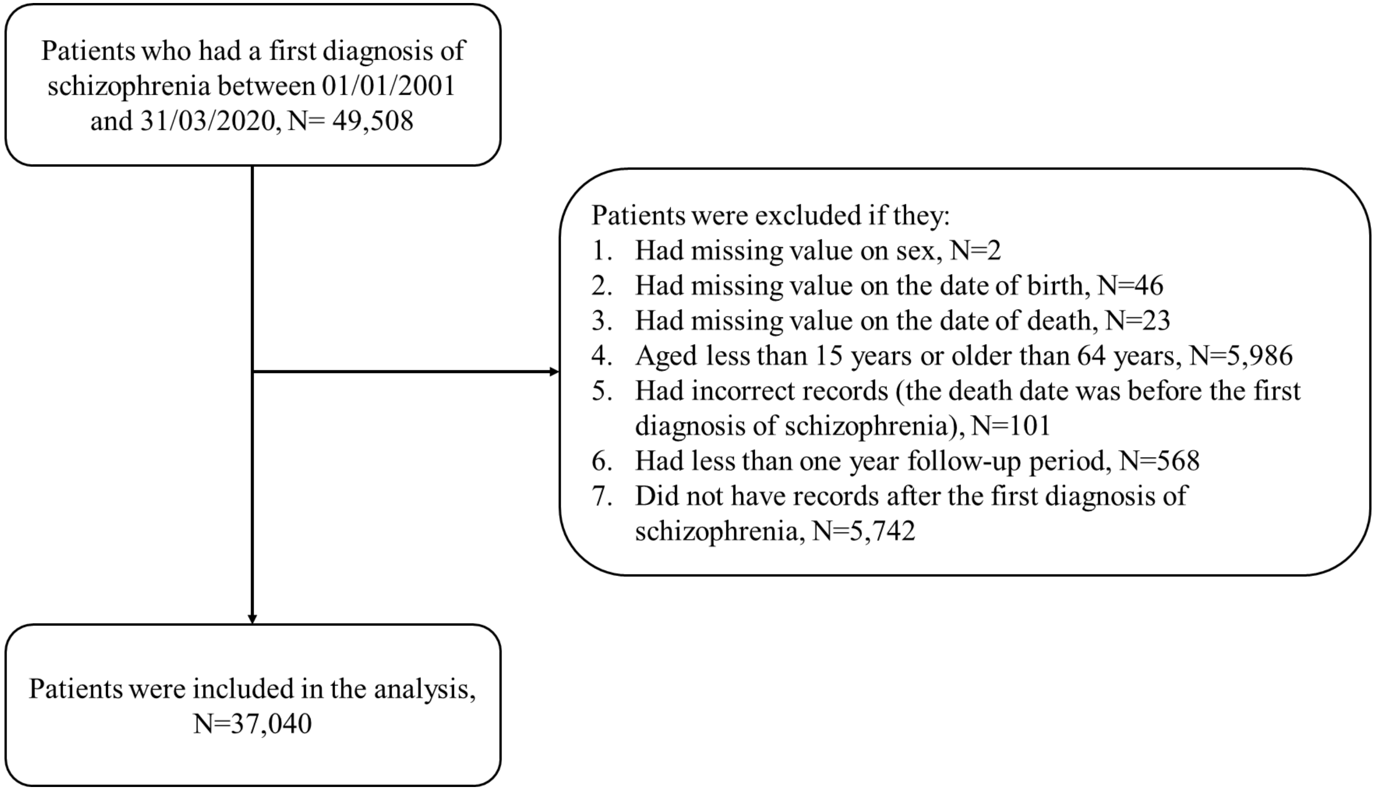

eFigure 1. Flowchart of the Study Sample Selection

© 2024 Chai Y et al. *JAMA Network Open.*

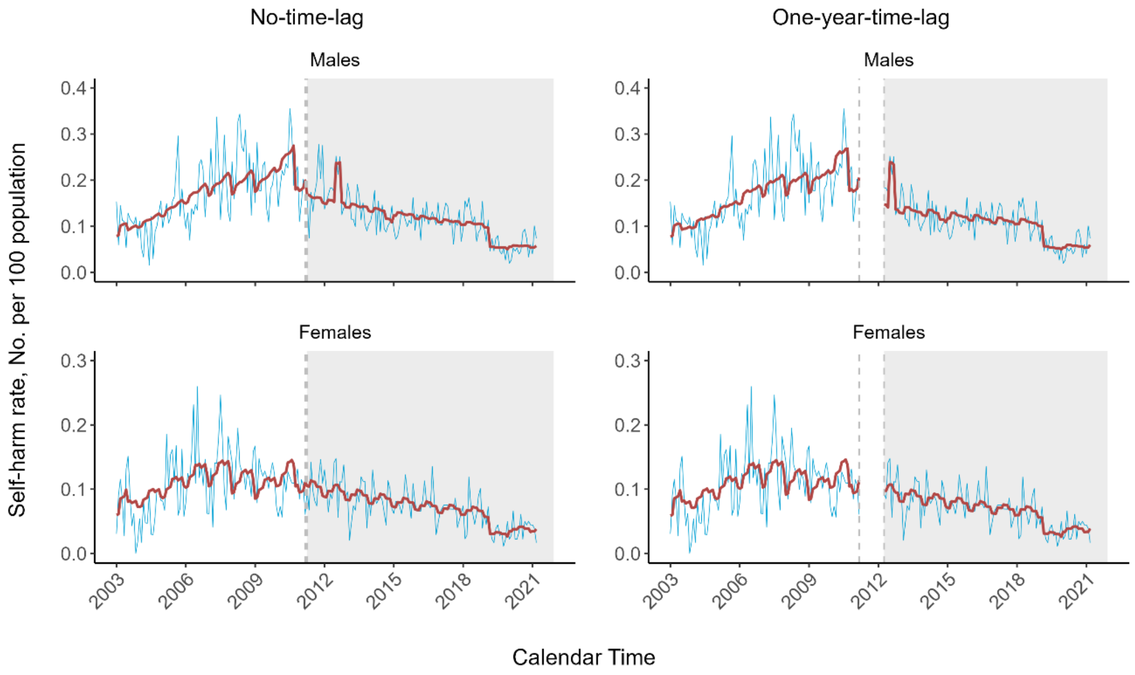

eFigure 2. Interrupted Time Series Analysis of Changes in Self-Harm Rates by Sex Group Before and After the Implementation of the Early Assessment Service for Young People With Early Psychosis for Patients Aged 15 to 64 Years (EASY Plus) Program

Blue lines indicate the observed self-harm rates

Red lines indicate the fitted self-harm rates based on the interrupted time series model

Gray areas indicate the EASY Plus intervention time

Vertical dashed lines in the no time lag analysis indicate March and April 2011

Vertical dashed lines in the one-year time lag analysis indicate March 2011 and March 2012

© 2024 Chai Y et al. *JAMA Network Open.*

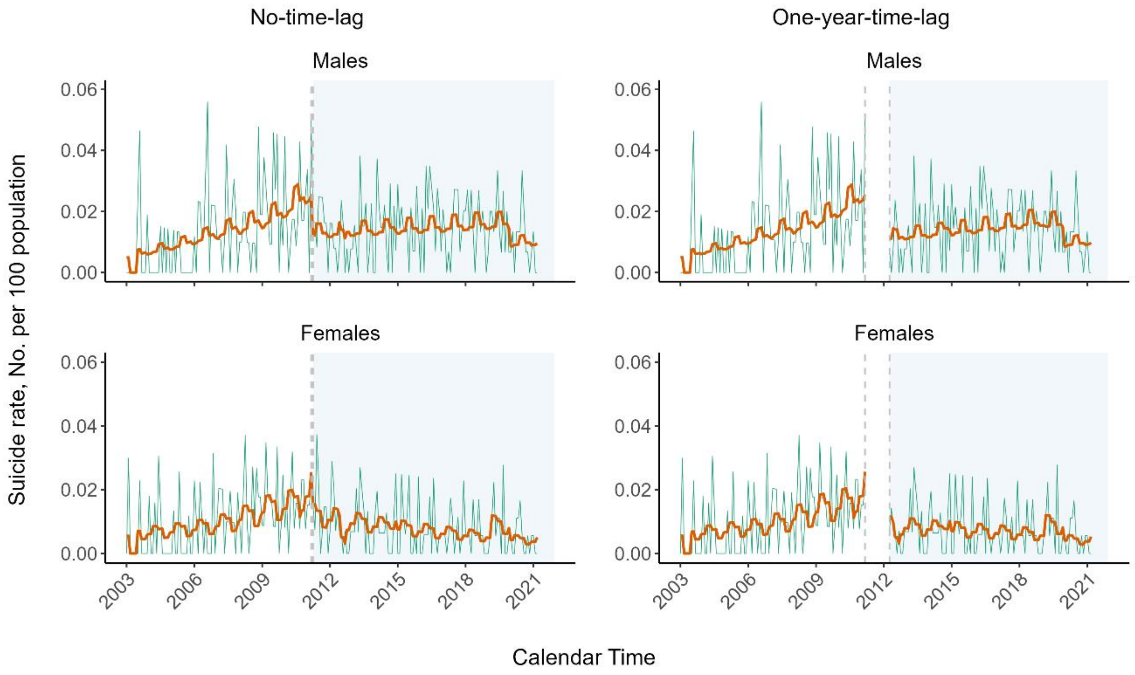

eFigure 3. Interrupted Time Series Analysis of Changes in Suicide Rates by Sex Group Before and After the Implementation of the Early Assessment Service for Young People With Early Psychosis for Patients Aged 15 to 64 Years (EASY Plus) Program

Green lines indicate the observed suicide rates

Orange lines indicate t the fitted suicide rates based on the interrupted time series model

Blue areas indicate the EASY Plus intervention time

Vertical dashed lines in the no time lag analysis indicate March and April 2011

Vertical dashed lines in the one-year time lag analysis indicate March 2011 and March 2012

© 2024 Chai Y et al. *JAMA Network Open.*

eTable 1. Full Results From Interrupted Time Series Analyses of Self-Harm Rates for the No-Time-Lag Analysis

	RR (95% CI)				
	**15-25 years**	**26-44 years**	**45-64 years**	**Males**	**Females**
**Background trend**	1.015 (1.001-1.029)	1.020 (1.010-1.031)	1.027 (1.012-1.042)	1.021 (1.012-1.031)	1.020 (1.009-1.031)
**Level change**	0.992 (0.649-1.519)	0.837 (0.647-1.082)	0.835 (0.589-1.184)	0.829 (0.652-1.054)	0.926 (0.691-1.242)
**Slope change**	0.982 (0.969-0.995)	0.976 (0.967-0.985)	0.968 (0.954-0.981)	0.975 (0.967-0.984)	0.977 (0.967-0.987)
**ICCMW**	0.519 (0.337-0.800)	0.703 (0.537-0.920)	0.768 (0.529-1.113)	0.642 (0.500-0.824)	0.736 (0.541-1.000)
**Social unrest events**					
Absence	ref	ref	ref	ref	ref
The social unrest event in 2012	1.651 (1.092-2.498)	1.211 (0.888-1.652)	1.294 (0.849-1.973)	1.568 (1.203-2.043)	1.027 (0.717-1.472)
The social unrest event in 2014	0.769 (0.460-1.285)	0.973 (0.716-1.321)	0.925 (0.588-1.455)	0.865 (0.634-1.181)	1.005 (0.722-1.399)
The social unrest event in 2019	0.595 (0.392-0.904)	0.462 (0.336-0.635)	0.567 (0.352-0.913)	0.543 (0.406-0.727)	0.486 (0.345-0.684)
**Infectious disease epidemics**					
Absence	ref	ref	ref	ref	ref
SARS	0.689 (0.245-1.936)	1.160 (0.601-2.236)	1.832 (0.717-4.682)	1.157 (0.603-2.223)	1.233 (0.617-2.463)
COVID-19	0.503 (0.333-0.758)	0.694 (0.537-0.895)	0.523 (0.333-0.823)	0.598 (0.461-0.777)	0.629 (0.471-0.839)
**Inpatient psychiatric beds per 100,000 people**	1.040 (0.995-1.087)	1.045 (1.012-1.080)	1.064 (1.016-1.115)	1.039 (1.008-1.070)	1.058 (1.021-1.096)
**Seasonal factor**					
Spring	ref	ref	ref	ref	ref
Summer	1.032 (0.866-1.229)	1.048 (0.930-1.182)	1.002 (0.839-1.197)	0.983 (0.878-1.102)	1.197 (0.961-1.251)
Autumn	1.012 (0.846-1.210)	0.994 (0.880-1.124)	1.046 (0.875-1.249)	0.998 (0.890-1.119)	1.030 (0.899-1.179)
Winter	0.901 (0.751-1.081)	0.921 (0.814-1.042)	0.950 (0.793-1.137)	0.941 (0.839-1.056)	0.900 (0.784-1.033)

RR: Rate ratio; 95% CI: 95% confidence interval; ICCMW: Integrated Community Centre for Mental Wellness program; The social unrest event in 2012: the Moral and National Education Controversy; The social unrest event in 2014: the Umbrella Movement; The social unrest event in 2019: the Anti-extradition Law Amendment Bill Movement.

© 2024 Chai Y et al. *JAMA Network Open.*

eTable 2. Full Results From Interrupted Time Series Analyses of Self-Harm Rates for the One-Year-Time-Lag Analysis

	RR (95% CI)				
	**15-25 years**	**26-44 years**	**45-64 years**	**Males**	**Females**
**Background trend**	1.015 (1.001-1.028)	1.020 (1.010-1.031)	1.027 (1.013-1.042)	1.021 (1.012-1.031)	1.020 (1.009-1.031)
**Level change**	0.863 (0.561-1.327)	0.768 (0.588-0.999)	0.703 (0.490-0.997)	0.714 (0.560-0.911)	0.868 (0.641-1.175)
**Slope change**	0.984 (0.971-0.997)	0.977 (0.967-0.986)	0.969 (0.955-0.983)	0.977 (0.968-0.985)	0.977 (0.967-0.987)
**ICCMW**	0.528 (0.345-0.809)	0.709 (0.540-0.930)	0.772 (0.533-1.118)	0.644 (0.505-0.823)	0.742 (0.545-1.012)
**Social unrest events**					
Absence	ref	ref	ref	ref	ref
The social unrest event in 2012	1.769 (1.150-2.722)	1.244 (0.898-1.722)	1.407 (0.905-2.189)	1.707 (1.299-2.244)	1.036 (0.711-1.508)
The social unrest event in 2014	0.809 (0.485-1.349)	0.997 (0.730-1.363)	0.993 (0.630-1.567)	0.913 (0.672-1.242)	1.020 (0.728-1.428)
The social unrest event in 2019	0.563 (0.370-0.858)	0.453 (0.327-0.627)	0.536 (0.331-0.868)	0.512 (0.383-0.684)	0.481 (0.339-0.684)
**Infectious disease epidemics**					
Absence	ref	ref	ref	ref	ref
SARS	0.672 (0.243-1.861)	1.148 (0.593-2.224)	1.789 (0.703-4.549)	1.137 (0.601-2.151)	1.213 (0.604-2.434)
COVID-19	0.470 (0.308-0.718)	0.676 (0.516-0.886)	0.484 (0.303-0.773)	0.553 (0.424-0.721)	0.625 (0.460-0.847)
**Inpatient psychiatric beds per 100,000 people**	1.040 (0.995-1.086)	1.046 (1.012-1.081)	1.066 (1.018-1.117)	1.039 (1.009-1.070)	1.058 (1.022-1.096)
**Seasonal factor**					
Spring	ref	ref	ref	ref	ref
Summer	1.005 (0.841-1.201)	1.041 (0.919-1.180)	0.974 (0.811-1.170)	0.966 (0.861-1.084)	1.081 (0.943-1.240)
Autumn	0.985 (0.821-1.181)	0.964 (0.848-1.096)	0.962 (0.798-1.158)	0.949 (0.844-1.067)	1.000 (0.869-1.152)
Winter	0.844 (0.700-1.017)	0.899 (0.790-1.023)	0.900 (0.747-1.084)	0.904 (0.804-1.017)	0.865 (0.749-0.999)

RR: Rate ratio; 95% CI: 95% confidence interval; ICCMW: Integrated Community Centre for Mental Wellness program; The social unrest event in 2012: the Moral and National Education Controversy; The social unrest event in 2014: the Umbrella Movement; The social unrest event in 2019: the Anti-extradition Law Amendment Bill Movement.

© 2024 Chai Y et al. *JAMA Network Open.*

eTable 3. Full Results From the Interrupted Time Series Analysis of Suicide Rates for the No-Time-Lag Analysis

	RR (95% CI)				
	**15-25 years**		**15-25 years**		**15-25 years**
**Background trend**	1.007 (0.967-1.049)	1.007 (0.979-1.035)	1.041 (0.990-1.0994)	1.020 (0.991-1.049)	1.007 (0.975-1.039)
**Level change**	0.506 (0.233-1.099)	0.466 (0.255-0.851)	0.749 (0.265-2.121)	0.471 (0.261-0.850)	0.614 (0.321-1.174)
**Slope change**	0.984 (0.947-1.022)	0.995 (0.969-1.021)	0.960 (0.916-1.006)	0.983 (0.957-1.010)	0.984 (0.955-1.014)
**ICCMW**	1.310 (0.552-3.108)	1.143 (0.605-2.160)	0.661 (0.207-2.106)	1.002 (0.531-1.892)	1.173 (0.573-2.400)
**Social unrest events**					
Absence	ref	ref	ref	ref	ref
The social unrest event in 2012	0.544 (0.129-2.294)	0.857 (0.313-2.350)	0.440 (0.056-3.470)	0.852 (0.337-2.153)	0.425 (0.100-1.795)
The social unrest event in 2014	0.313 (0.043-2.298)	1.732 (0.944-3.176)	0.472 (0.061-3.676)	1.050 (0.504-2.188)	1.306 (0.551-3.095)
The social unrest event in 2019	1.591 (0.711-3.559)	0.988 (0.581-1.679)	1.541 (0.686-3.461)	0.981 (0.595-1.618)	1.777 (0.939-3.365)
**Infectious disease epidemics**					
Absence	ref	ref	ref	ref	ref
SARS	-	-	-	-	-
COVID-19	1.095 (0.445-2.696)	0.614 (0.339-1.112)	0.547 (0.192-1.558)	0.576 (0.328-1.013)	0.957 (0.445-2.055)
**Inpatient psychiatric beds per 100,000 people**	0.973 (0.853-1.110)	0.987 (0.902-1.079)	1.080 (0.923-1.263)	1.018 (0.929-1.115)	0.980 (0.886-1.085)
**Seasonal factor**					
Spring	ref	ref	ref	ref	ref
Summer	1.427 (0.942-2.161)	1.065 (0.786-1.444)	0.867 (0.557-1.349)	1.310 (0.980-1.752)	0.880 (0.633-1.223)
Autumn	0.839 (0.523-1.344)	1.002 (0.737-1.363)	0.433 (0.249-0.751)	1.047 (0.771-1.422)	0.556 (0.383-0.807)
Winter	0.923 (0.588-1.450)	0.888 (0.969-1.021)	0.679 (0.422-1.092)	0.948 (0.695-1.292)	0.722 (0.514-1.014)

RR: Rate ratio; 95% CI: 95% confidence interval; ICCMW: Integrated Community Centre for Mental Wellness program; The social unrest event in 2012: the Moral and National Education Controversy; The social unrest event in 2014: the Umbrella Movement; The social unrest event in 2019: the Anti-extradition Law Amendment Bill Movement.

© 2024 Chai Y et al. *JAMA Network Open.*

eTable 4. Full Results From the Interrupted Time Series Analysis of Suicide Rates for the One-Year-Time-Lag Analysis

	**RR (95% CI)**				
	**15-25 years**	**26-44 years**	**45-64 years**	**Males**	**Females**
**Background trend**	1.007 (0.966-1.049)	1.006 (0.978-1.034)	1.043 (0.991-1.097)	1.020 (0.990-1.050)	1.007 (0.976-1.039)
**Level change**	0.333 (0.144-0.770)	0.384 (0.203-0.727)	0.775 (0.269-2.230)	0.429 (0.230-0.801)	0.446 (0.225-0.884)
**Slope change**	0.990 (0.951-1.029)	0.999 (0.973-1.026)	0.957 (0.712-1.004)	0.986 (0.958-1.014)	0.988 (0.959-1.018)
**ICCMW**	1.312 (0.549-3.134)	1.130 (0.594-2.153)	0.661 (0.207-2.109)	1.003 (0.523-1.921)	1.168 (0.572-2.304)
**Social unrest events**					
Absence	ref	ref	ref	ref	ref
The social unrest event in 2012	0.741 (0.167-3.287)	1.079 (0.376-3.092)	0.414 (0.051-3.341)	0.970 (0.365-2.577)	0.534 (0.124-2.304)
The social unrest event in 2014	0.380 (0.051-2.837)	1.900 (1.012-3.569)	0.465 (0.059-3.642)	1.126 (0.526-2.411)	1.439 (0.601-3.445)
The social unrest event in 2019	1.294 (0.564-2.969)	0.879 (0.506-1.525)	1.601 (0.688-3.725)	0.917 (0.542-1.552)	1.552 (0.804-2.994)
**Infectious disease epidemics**					
Absence	ref	ref	ref	ref	ref
SARS	-	-	-	-	-
COVID-19	0.829 (0.322-2.135)	0.517 (0.276-0.968)	0.583 (0.195-1.745)	0.520 (0.284-0.949)	0.791 (0.356-1.755)
**Inpatient psychiatric beds per 100,000 people**	0.973 (0.853-1.110)	0.984 (0.898-1.077)	1.088 (0.929-1.274)	1.018 (0.927-1.117)	0.980 (0.886-1.085)
**Seasonal factor**					
Spring	ref	ref	ref	ref	ref
Summer	1.385 (0.896-2.140)	1.165 (0.772-1.470)	0.823 (0.522-1.297)	1.295 (0.953-1.760)	0.839 (0.594-1.185)
Autumn	0.751 (0.452-1.247)	1.003 (0.724-1.388)	0.421 (0.239-0.741)	1.012 (0.731-1.401)	0.532 (0.359-0.786)
Winter	0.910 (0.567-1.460)	0.935 (0.675-1.295)	0.607 (0.368-0.999)	0.949 (0.685-1.315)	0.711 (0.500-1.012)

RR: Rate ratio; 95% CI: 95% confidence interval; ICCMW: Integrated Community Centre for Mental Wellness program; The social unrest event in 2012: the Moral and National Education Controversy; The social unrest event in 2014: the Umbrella Movement; The social unrest event in 2019: the Anti-extradition Law Amendment Bill Movement.

© 2024 Chai Y et al. *JAMA Network Open.*

eTable 5. Estimates of the Level and Slope Changes in Self-Harm and Suicide Rates From the Two-Year-Time-Lag Analysis and Newey-West Standard Errors Corrected Analysis

	**Two-year-time-lag analysis**		**Newey-West standard errors correction (No-time-lag)**		**Newey-West standard errors correction (One-year-time-lag)**	
	**Level change, RR (95% CI)**	**Slope change, RR (95% CI)**	**Level change, RR (95% CI)**	**Slope change, RR (95% CI)**	**Level change, RR (95% CI)**	**Slope change, RR (95% CI)**
**Self-harm**						
15-25 years	0.746 (0.481-1.156)	0.986 (0.973-1.000)	0.993 (0.792-1.245)	0.982 (0.971-0.993)	0.866 (0.701-1.071)	0.983 (0.973-0.994)
26-44 years	0.750 (0.572-0.984)	0.976 (0.966-0.986)	0.839 (0.728-0.968)	0.977 (0.966-0.988)	0.767 (0.670-0.878)	0.978 (0.967-0.989)
45-64 years	0.525 (0.364-0.758)	0.973 (0.960-0.987)	0.832 (0.638-1.085)	0.969 (0.954-0.984)	0.691 (0.513-0.931)	0.970 (0.953-0.988)
Males	0.614 (0.478-0.790)	0.979 (0.970-0.988)	0.831 (0.668-1.034)	0.975 (0.966-0.985)	0.710 (0.574-0.877)	0.977 (0.967-0.987)
Females	0.819 (0.606-1.108)	0.978 (0.967-0.988)	0.923 (0.794-1.073)	0.978 (0.964-0.992)	0.862 (0.712-1.043)	0.978 (0.964-0.992)
**Suicide**						
15-25 years	0.337 (0.144-0.788)	0.987 (0.952-1.023)	0.547 (0.250-1.196)	0.978 (0.945-1.012)	0.547 (0.250-1.196)	0.978 (0.945-1.012)
26-44 years	0.529 (0.279-1.001)	0.990 (0.965-1.016)	0.474 (0.255-0.881)	0.998 (0.968-1.030)	0.385 (0.235-0.631)	1.003 (0.976-1.031)
45-64 years	1.109 (0.375-3.280)	0.994 (0.957-1.033)	1.156 (0.742-1.802)	1.004 (0.969-1.041)	1.154 (0.752-1.772)	1.001 (0.965-1.038)
Males	0.597 (0.319-1.119)	0.984 (0.959-1.010)	0.533 (0.388-0.732)	0.991 (0.964-1.019)	0.495 (0.261-0.938)	0.993 (0.969-1.018)
Females	0.508 (0.250-1.035)	0.994 (0.966-1.022)	0.701 (0.465-1.057)	0.995 (0.974-1.016)	0.508 (0.320-0.804)	0.999 (0.977-1.021)

RR: Rate ratio; 95% CI: 95% confidence interval

© 2024 Chai Y et al. *JAMA Network Open.*

eTable 6. Estimates of the Level and Slope Changes in Self-Harm and Suicide Rates From the Stricter Definition of Self-Harm and Suicide Analysis

	Stricter definition of self-harm and suicide (No-time-lag)		Stricter definition of self-harm and suicide (One-year-time-lag)	
	**Level change, RR (95% CI)**	**Slope change, RR (95% CI)**	**Level change, RR (95% CI)**	**Slope change, RR (95% CI)**
**Self-harm**				
15-25 years	1.486 (0.838-2.634)	0.985 (0.971-0.999)	1.550 (0.863-2.783)	0.984 (0.970-0.999)
26-44 years	1.050 (0.765-1.442)	0.980 (0.970-0.990)	1.020 (0.737-1.411)	0.980 (0.969-0.990)
45-64 years	0.820 (0.507-1.326)	0.975 (0.958-0.992)	0.549 (0.520-1.387)	0.974 (0.957-0.992)
Males	1.124 (0.813-1.556)	0.982 (0.972-0.991)	1.168 (0.840-1.624)	0.981 (0.971-0.991)
Females	0.986 (0.700-1.390)	0.979 (0.968-0.990)	0.949 (0.665-1.354)	0.979 (0.967-0.991)
**Suicide**				
15-25 years	0.479 (0.222-1.033)	0.985 (0.948-1.023)	0.329 (0.143-0.754)	0.990 (0.952-1.030)
26-44 years	0.459 (0.243-0.867)	0.982 (0.954-1.010)	0.378 (0.192-0.743)	0.986 (0.958-1.016)
45-64 years	0.694 (0.241-1.994)	0.960 (0.915-1.006)	0.690 (0.236-2.023)	0.957 (0.912-1.005)
Males	0.438 (0.243-0.789)	0.977 (0.951-1.005)	0.401 (0.215-0.749)	0.980 (0.952-1.008)
Females	0.619 (0.316-1.215)	0.976 (0.946-1.007)	0.445 (0.218-0.909)	0.980 (0.949-1.012)

RR: Rate ratio; 95% CI: 95% confidence interval

© 2024 Chai Y et al. *JAMA Network Open.*
