# Supplementary material for: Self-Harm and Suicide Rates Before and After an Early Intervention Program for Patients With First-Episode Schizophrenia
Source: JAMA Netw Open. 2024 Aug 8;7(8):e2426795. doi: 10.1001/jamanetworkopen.2024.26795 (PMC11310822; doi:10.1001/jamanetworkopen.2024.26795)
Supplement: Supplement 2. — Data Sharing Statement [file jamanetwopen-e2426795-s002.pdf]

Data Sharing Statement

Chai. Self-Harm and Suicide Rates Before and After an Early Intervention Program for Patients With First-Episode Schizophrenia. *JAMA Netw Open*. Published August 08, 2024. doi:10.1001/jamanetworkopen.2024.26795

Data
Data available: No

Additional Information
Explanation for why data not available: The data is part of the registry data and cannot be made available publicly.
